# Part II – imidazolines and rhinitis medicamentosa: how can we tackle the rebound dilemma?

**DOI:** 10.3389/fphar.2025.1655254

**Published:** 2025-11-28

**Authors:** Rebecca J. Stinson, Laura R. Sadofsky

**Affiliations:** Biomedical Institute for Multimorbidity, Centre for Biomedicine, Hull York Medical School, University of Hull, Hull, United Kingdom

**Keywords:** nasal decongestants, imidazolines, oxymetazoline, sympathomimetic, nasal decongestants/adverse effects, imidazoline receptor, rhinitis medicamentosa, rebound

## Abstract

Rhinitis medicamentosa is the state of chronic congestion experienced from the prolonged or excessive use of nasal vasoconstrictors, typically used in the treatment of nasal congestion. Although a minor ailment, nasal congestion is a bothersome symptom for many allergic and non-allergic rhinitis sufferers. To alleviate symptoms, patients turn to over-the-counter topical nasal decongestants, with imidazolines often being the active ingredient of choice, as they provide rapid and long-lasting symptom relief, via vasoconstriction within the nasal mucosa. When used correctly, nasal vasoconstrictors are considered safe. However, misuse and deviation away from the recommended frequency of dose or maximum length of treatment can result initially in acute rebound congestion and if continued, rhinitis medicamentosa (RM). The pathophysiology and mechanisms of RM have not been fully elucidated and are complicated by difficulties in determining where the underlying disease ends, and RM begins. RM is characterised by the presence of chronic nasal congestion which responds less effectively and for progressively shorter periods to nasal vasoconstrictor treatments. Consequently, patients try to relieve symptoms by taking the nasal vasoconstrictor more frequently or at higher doses, which ultimately exacerbates the congestion further and creates a dependency on the nasal vasoconstrictor. Why RM develops is unclear, hypothesised mechanisms include structural, functional and inflammatory changes in the nasal mucosa, changes in receptor sensitivity or innervation pathways. To which extent this is solely in response to the use of imidazoline derivates or related to the presence of preservatives in topical nasal treatments is yet to be determined. Although treatment options exist for RM, prevention may be the best medicine. Provision of better education around the use of nasal vasoconstrictions, coupled with preservative free formulations and lowest possible dose delivery, may help to reduce the likelihood of developing the disease from the outset, reducing the burden on both the patient and healthcare providers.

## 1 Introduction

Imidazolines were first synthesised in the 1880s however, they have only been utilised therapeutically since the early 1940s. Imidazolines have a wide range of therapeutic uses but are perhaps most widely recognised as potent nasal vasoconstrictors and anti-hypertensives (Krasavin, 2015; Mehedi and Tepe, 2020; National Center for Biotechnology Information). In part one of our review series, we considered the history of the imidazolines, their receptor interactions, efficacy and tolerability, particularly in relation to nasal vasoconstrictors. Imidazoline derived nasal vasoconstrictors typically have a relatively low risk of causing adverse side effects when used correctly however, if misused the risk of adverse events increases (Black and Remsen, 1980; Trinh et al., 2024; Wang et al., 2024). Thus, the focus of this review is the potential issues and risk surrounding the prolonged use of imidazoline nasal vasoconstrictors, especially that of rhinitis medicamentosa (RM). Why RM develops and how it can be managed is a subject of much debate and the literature surrounding this topic is confusing. In this literature review we focus on the role imidazoline derived nasal vasoconstrictors play in RM and aim to unpick some of this puzzle, considering the pathophysiology of the disease, potential treatment and most importantly its prevention. The reason for commencing the use of imidazoline nasal vasoconstrictors is typically due to experiencing prolonged episodes of nasal congestion (Wahid, 2023). Nasal congestion is a troublesome symptom in numerous conditions including allergic and non-allergic rhinitis, infections, nasal polyps, sinusitis, and as a side effect of some medications (Craig et al., 2010; Stewart et al., 2010). Due to the differing aetiologies, estimating the number of people who are suffering with nasal congestion at any given time is difficult (Table 1). However, given that nasal congestion is experienced by approximately 30% of allergic rhinitis and 90% of non-allergic rhinitis sufferers, the burden of this symptom is widely felt (Bernstein et al., 2024; Savouré et al., 2022; Bhargava et al., 2011). Whilst nasal congestion is in itself a minor ailment, the impact on the sufferer can extend beyond a stuffy nose to include a range of other symptoms (Table 1) (Stewart et al., 2010). Nasal decongestants can be grouped based on their active pharmacological ingredient (API) and as such their mechanism of action. There are four main types widely available, sympathomimetic topical and oral preparations, i.e., β-phenylethylamine and imidazoline derivatives, saline nasal irrigations, corticosteroid nasal sprays and antihistamine nasal sprays (Wang et al., 2024; Wahid, 2023; Berlin et al., 2000; Li et al., 2019; Hermelingmeier et al., 2012). These can contain more than one API, which increases the potential for confusion for the patient. Furthermore, nasal decongestants are potentially viewed as harmless by patients, due to the assumption that ready availability without prescription equates to a low dose formulation. Thus patients may underestimate the potential risk of experiencing adverse effects if recommended dosage or treatment length is not followed (Trinh et al., 2024). This literature review will focus on the sympathomimetic nasal decongestants, specifically the imidazoline derivatives, which when used correctly are safe and produce minimal side effects. However, prolonged use of these nasal vasoconstrictors can increase the risk of developing RM. This chronic sensation of nasal congestion is associated with the prolonged and excessive use of any sympathomimetic nasal decongestants (Russo et al., 2023). Even when used at the correct duration and frequency, patients can experience rebound congestion immediately after ceasing their use, but unlike RM, this short-term acute congestion typically resolves quickly and without any additional treatment (Black and Remsen, 1980; Mortuaire et al., 2013; Lockey, 2006).

**TABLE 1:** Estimated prevalence of differing aetiologies of rhinitis and the extent to which nasal congestion is experienced. Patients experiencing nasal congestion, regardless of aetiology, may also experience a range of additional symptoms (Stewart et al., 2010; Bernstein et al., 2024; Savouré et al., 2022; Bhargava et al., 2011).

Aetiology of rhinitis	Estimated median worldwide prevalence	Percentage experiencing nasal congestion
Allergic	12%	30%
Non-allergic	18%	90%
Unspecified	30%	Undetermined
Additional symptoms of nasal congestion	Headaches	
	Face and ear pressure/pain	
	Rhinorrhoea	
	Postnasal drip	
	Snoring and sleep disturbances	
	Fatigue	
	Loss of smell and taste	
	Bad breath	

## 2 Unpicking the rhinitis medicamentosa puzzle

### 2.1 Rebound vs. rhinitis medicamentosa

Prolonged use of topical imidazolines has frequently been linked to the phenomenon known as rebound congestion, rhinitis medicamentosa or drug-induced rhinitis. Determining whether these terms represent the exact same condition or a group of similar conditions which marginally differ in clinical presentation is problematic, as too is providing a clear definition. This issue arises due to lack of clarity around whether these terms all describe the same pathophysiological effect, clinical diagnostic criteria being vague and the interchangeable use of the terms (Mortuaire et al., 2013). First described in the mid-1940s, rebound congestion is often used to describe the experience of continued nasal congestion after stopping nasal decongestive treatment. RM could be considered a syndrome of rebound congestion. It describes the persistent presence of nasal congestion, which is relieved for progressively shorter periods of time after treatment. It is typically experienced by individuals who have a history of prolonged use of nasal decongestants and find more frequent or increase dosages are required to relieve symptoms. Whilst these phenomena are clearly related due to the similarity of symptoms and clear causal link to nasal decongestants, it is reasonable to consider one as acute and the other chronic (Table 2) (Mortuaire et al., 2013; Ramey et al., 2006; Wallace et al., 2008; Scadding, 1995; Graf, 1999).

**TABLE 2:** Differences between rebound congestion and rhinitis medicamentosa which can result from the misuse or prolonged use of imidazoline based nasal treatments, the effects can be dependent on the API and formulation. Acute rebound congestion phase can progress to chronic rhinitis medicamentosa phase if nasal vasoconstrictor usage is not curtailed (Black and Remsen, 1980; Mortuaire et al., 2013; Lockey, 2006; Ramey et al., 2006; Wallace et al., 2008; Scadding, 1995; Graf, 1999; Graf and Juto, 1994a; Graf and Juto, 1995; Knipping et al., 2007; Lin et al., 2004; Zheng et al., 2025).

Potential impact of treatment misuse		Rebound congestion	Rhinitis medicamentosa
Type of congestion		Acute	Chronic
Symptoms		Worsening congestion	Persistent congestion
Pathophysiology		No change	Changes to nasal structure, innervation, immunological response and receptor sensitivity
Dosage volume/frequency of dose being used		Recommended	Excessive
Duration of nasal vasoconstrictor use		Days to weeks	Weeks to years
Occurrence	After type of use	Misuse	Prolonged use
	When during use	Within 24 h of stopping	During usage
Resolution of symptoms without treatment		1–2 weeks	Unlikely
Managed withdrawal of nasal decongestant required		No	Yes
Treatment	Medical interventions required	None	Intranasal glucocorticosteroids Saline nasal washes Oral steroids
	Surgical intervention required	No	Possibly

Rebound congestion is experienced shortly after the vasoconstrictive effect of the treatment subsides or post cessation of short-term nasal vasoconstrictor treatment and typically resolves within 1–2 weeks of discontinuing treatment. However, this acute rebound congestion can be bothersome for some patients. Consequently they continue to use the nasal vasoconstrictor which further exacerbates the nasal congestion leading to the development of RM. Thus, RM is suggestive of a chronic effect experienced after prolonged use of nasal vasoconstrictors over a period of several weeks to months. Treatment which aims to reverse the effects of RM needs careful consideration, as sudden withdrawal may create a rebound effect which further exacerbates the symptoms being experienced. Thus, slow withdrawal coupled with alternative treatment such as saline nasal washes, intranasal glucocorticosteroids or if necessary oral steroids, is the most effective way of resolving the patient’s chronic nasal congestion (Table 2) (Black and Remsen, 1980; Lockey, 2006; Graf, 1999). RM is not exclusive to topical nasal decongestants and can be a result of using oral medications such as antihypertensives, β-adrenoreceptor antagonists, oral contraceptives, and antipsychotics (Ramey et al., 2006). For example, sudden discontinuation of the imidazolines derived hypertensive drug clonidine, results in a rebound hypertension, with readings often higher than seen pre-treatment, thus tapered withdrawal is required (Met et al., 1987; Parkin et al., 2003; Rupp et al., 1996; Schäfer et al., 1995; Ollivier and Christen, 1994). Since these treatments are used orally, the mechanisms involved in the development of RM may differ to that involved in topically applied medications. Thus this side effect is more frequently referred to as drug-induced rhinitis although often the symptoms experienced are the same (Ramey et al., 2006). Given the lack of a clear definition of either term it is understandable why they have become interchangeable and generally synonymous with the misuse of nasal vasoconstrictors. However, regardless of the name used to describe this phenomenon, the symptoms experienced have a high degree of similarity and are often as bothersome, if not more so than the initial symptoms that were being treated by the nasal decongestant. Thus, developing a clearer understanding of the pathophysiology which occurs during acute rebound congestion or chronic RM, may help provide a more definitive definition, which could subsequently aid in the clinical diagnosis and optimise treatment pathways.

The literature around RM is somewhat contradictory due to the subjective nature of the disorder experienced by patients and the evidence available. Some studies have shown that changes in nasal patency and histology, alongside oedema of the nasal mucosa can result from prolonged treatment with certain nasal vasoconstrictors (Graf and Juto, 1994a; Graf and Juto, 1995; Knipping et al., 2007; Lin et al., 2004). Difficulty in finding evidence to support the presence of RM is in part owing to the overlap between symptoms of RM and the underlying disease which resulted in the commencement of nasal vasoconstrictor, furthermore not all individuals who use nasal vasoconstrictors for prolonged periods experience RM especially when recommended dosing is followed. Other issues pertain to studies either being done *in vitro*, in animal model or *in vivo* on healthy human subjects, thus the underlying physiological changes that are experienced in the diseased tissue are not present in many instances (Mortuaire et al., 2013; Margulis et al., 2024; Graf et al., 1999; Eccles et al., 2008). Ultimately, this lack of clarity in existing evidence makes the creation of a definitive diagnostic criteria for RM difficult. Additionally, clinical findings are often non-specific owing to the overlap between RM and other causative reasons for nasal congestion. Without any specific biochemical test, determining whether an individual has RM is based on patient examination and history, including their use of nasal vasoconstrictors (Wahid, 2023; Ramey et al., 2006; Zucker et al., 2019; Doshi, 2009). Thus, there is the need to create a uniformed approach to diagnostics, which would allow for clear differentiation between acute rebound congestion and chronic RM. A standardized questionnaire for patient history may go some way to help with this process. This could enable multi-centre studies to be conducted to assess patterns in patient presentation and further clarify diagnostic criteria. Additionally, consistent use of sinonasal endoscopy may help to provide further insight into how the nasal mucosa changes before and after the cessation of nasal vasoconstrictors (Zucker et al., 2019; Li et al., 2021; Fowler et al., 2019). However, whilst the pathophysiology and mechanisms of RM are not clearly understood, and uniformed diagnostic criteria remains lacking, clinical recommendations for the use of nasal vasoconstrictors remain at a maximum of 3–10 days, dependent on the API and formula, to minimise the risk of RM (Mortuaire et al., 2013; Margulis et al., 2024; Graf et al., 1999; Eccles et al., 2008).

### 2.2 Pathophysiology of rhinitis medicamentosa

RM is described as a type of non-allergic rhinitis which fits under the umbrella term of drug-induced rhinitis. It is characterised by localised inflammation of the nasal mucosa and hyperaemia, without the presence of sneezing, post-nasal drip or rhinorrhea (Black and Remsen, 1980; Ramey et al., 2006; Graf, 2005; Zheng et al., 2025). RM is most frequently observed in young to middle-aged adults, with similar levels of occurrence in males and females. It is estimated to account for 1%–9% of all visits to specialist clinics (Lockey, 2006). The sensation of stuffiness experience during RM is due to an increase in nasal resistance and changes in circadian nasal cycle, with one nostril often having a different extent of patency compared to the other. Based on this evidence, the continued congestion experienced by RM sufferers is not subjective, but rather a consequence of changes to nasal patency (Passàli et al., 2006). However, the exact mechanisms which underlies RM and results in these changes to nasal patency are not fully elucidated. Several potential mechanisms have been hypothesised relating to changes in innervation, receptor sensitivity and structural or inflammatory changes within the nasal mucosa (Ramey et al., 2006; Doshi, 2009; Graf, 2005).

**FIGURE 1:**
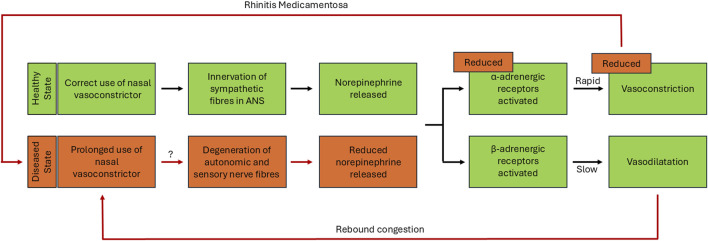
Hypothesised changes in innervation pathway during rhinitis medicamentosa. Representation of the sympathetic pathway leading to vasoconstriction and vasodilatation in healthy tissue and hypothesised changes within diseased tissue state. In the correct-use-phase, innervation of sympathetic nerve fibres within the nasal mucosa of healthy tissue results in the release of norepinephrine, which activates α- and β-adrenergic receptors leading to either vasoconstriction or vasodilation of the underlying nasal vasculature (shown in green). The vasodilatation which occurs after this correct-use-phase, can result in a rebound congestion driving the misuse of the vasoconstrictor, leading to the acute rebound phase (shown in green leading to red). If the use of nasal vasoconstrictors is further prolonged after this initial rebound phase, this finally results in the RM phase. The hypothesised changes to innervation pathway in the diseased state of rhinitis medicamentosa, causes a dysregulation in innervation, reducing norepinephrine release which leads to a reduction in the effectiveness of vasoconstriction provided by treatment (shown in red).

In healthy individuals, nasal mucosa innervation occurs via the sympathetic and parasympathetic fibres within the autonomic nervous system, providing homeostatic regulation and protective function against external chemical and physical stimuli (Sarin et al., 2006). The innervation of the sympathetic fibres releases norepinephrine which acts on either the α- or β-adrenergic receptors, resulting in vasoconstriction or vasodilatation respectively. Additionally, innervation of parasympathetic fibres and sensory C-fibres leads to changes in nasal secretions and reduced sympathetic tone, giving rise to the symptoms of congestion (Doshi, 2009). The diseased state experienced during RM may lead to changes within this innervation pathway. One hypothesis considers a negative feedback mechanism whereby production of endogenous sympathetic norepinephrine is reduced. Subsequently limiting the extent of vasoconstriction experienced when the nasal vasoconstrictor is discontinued or used for a prolonged period (Figure 1) (Ramey et al., 2006; Graf, 2005). Whilst true of the imidazolines which act on α-adrenergic receptors, it would not explain any decongestant which works predominantly on the β-adrenergic receptors (Zheng et al., 2025). An alternative hypothesis, suggests continual exposure to nasal vasoconstrictors could result in the degeneration of the autonomic and sensory nerve fibres linked to nasal congestion (Cauna and Cauna, 1974). The rebound congestion experienced could be a consequence of the initial activation of α-adrenergic receptors, resulting in a rapid decongestive effect being followed by a slower and prolonged activation of the β-adrenergic receptors, causing vasodilatation and the sensation of continued congestion (Doshi, 2009). The complex nature of RM means that there is potential for both the negative feedback loop and changes to innervation of the nasal mucosa hypotheses to play a role and are likely to be only part of the story. There is also some indication that histamine receptors may play a role in the development of RM. Imidazoline treated nasal epithelial cells have been shown to have increased mRNA expression of histamine H1 receptors *in vitro*, suggesting that this may further exacerbate RM symptoms, especially in patients with underlying allergic rhinitis. Importantly, this study also showed that treatment with the widely used preservative benzalkonium chloride (BKC) had a more significant impact on expression than the imidazoline treatment alone (Kawabata et al., 2016). Similar effects have been shown *in vivo* in healthy individuals who experienced an increased sensitivity to histamine after treatment over a 30-day period however, the role of BKC in these studies is less clear (Graf and Juto, 1994b; Graf, 1996; Graf PM. and Juto J-E., 1994). Whether the role that histamine receptors play in RM has any true significance is currently unclear. Furthermore, the presence of BKC in some formulas adds an additional layer of complexity to the findings. Nevertheless, the role histamine receptors play in RM would benefit further investigation in future studies (Figure 1).

Beyond the hypothesised mechanistic changes which may occur during RM, histological and immunological changes have also been identified. The changes associated with RM include modification of the epithelial layer, resulting in changes to cell structure and function such as increased mucus production, loss of ciliated cells and changes to cell type, alongside increased infiltration by immune cells (Table 3) (Ramey et al., 2006). It is worth noting that these observed changes have come through a mixture of study types, including both *in vitro* and *in vivo* human studies and animal modelling. As such it can be difficult to extrapolate how findings *in vitro* and in animal studies directly relate to pathological changes *in vivo*. Consequently, some of the histological and immunological changes discussed would benefit from further investigation *in vivo*, to better determine the role they play in RM.

**TABLE 3:** Histological and immunological changes observed within the nasal mucosa after treatment with imidazolines. Rhinitis medicamentosa studies on different animal tissues show various changes to the nasal mucosa based on species observed in (Ramey et al., 2006; Graf and Juto, 1994a; Graf and Juto, 1995; Knipping et al., 2007; Lin et al., 2004; Passàli et al., 2006; Elwany and Stephanos, 2010; Morissette et al., 2007; Suh et al., 1995; Talaat et al., 1981; Petruson and Hansson, 1982; De Corso et al., 2020; Wang and Bu, 1991).

Effect seen	Tissue type identified in		
	Human	Rabbit	Guinea pig
Damage and loss of ciliated cells	✓	✓	-
Increased permeability of basal lamina	✓	✓	-
Ulcerated epithelia	-	✓	-
Infiltration by inflammatory cells	-	✓	✓
Oedema	✓	✓	✓
Vacuolisation in endoplasmic reticulum, vesicles and mitochondria	-	✓	-
Cytopathic and cytotoxic	-	✓	-
Metaplasia of squamous cells	-	-	✓
Hyperplasia of goblet cells	✓	-	✓
Increased phagocytic activity	-	-	✓
Chronic inflammation	✓	-	-
Hypersecretion	✓	-	✓
Increased epidermal growth factors	✓	-	-
Increased interstitial gap	✓	-	-
Changes to vascular endothelial layer	✓	-	-
Increased vascularity	-	-	✓

**FIGURE 2:**
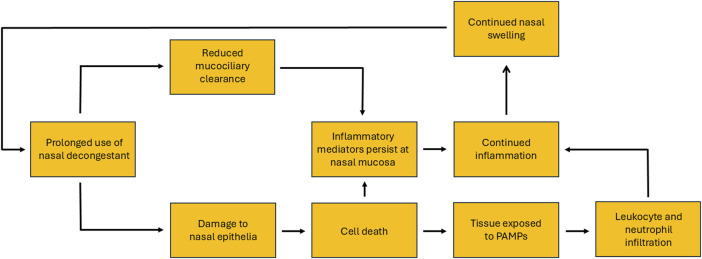
Proposed model of the inflammation-loop caused by rhinitis medicamentosa, initiated by the misuse of topical nasal vasoconstrictors and aggravated by their prolonged use.

In animal models there is convincing evidence that treatment with imidazoline based nasal formulas result in changes to the nasal mucosa, including increased permeability of the basal lamina, severe damage to ciliated cells and oedema (Ramey et al., 2006; Elwany and Stephanos, 2010; Morissette et al., 2007; Suh et al., 1995; Talaat et al., 1981). Studies in rabbits have shown evidence of ulcerated epithelia, infiltration by inflammatory cells and the presence of oedema, which becomes more pronounced as treatment continues. Additionally, there is also evidence of potential cytotoxic and cytopathic effects and vacuolisation. The latter may enable the sequestration of the decongestant into the tissue, driving osmotic swelling and resulting oedema (Morissette et al., 2007; Suh et al., 1995). Whilst studies in guinea pigs have shown evidence of metaplasia of squamous cells, hyperplasia of goblet cells, oedema, infiltration by mononuclear cells and increases in secretory, vascularity and phagocytic activity (Ramey et al., 2006; Elwany and Stephanos, 2010). The evidence of such changes in humans is less clear. Studies on healthy subjects who used xylometazoline for 6 weeks showed no changes in morphology or mucociliary function (Petruson and Hansson, 1982). However, this was partly contradicted in later studies using either xylometazoline or oxymetazoline. Although both nasal vasoconstrictors were shown to cause no evidence of rebound swelling after 10 days of treatment, by 30 days rebound congestion was experienced by most participants. Interestingly, the number of times daily the nasal vasoconstrictor was used did not influence the likelihood of developing rebound congestion. Whilst rebound swelling was observed after prolonged treatment in both studies, there was no consensus on whether the effect is a direct consequence of vasodilatation or interstitial oedema. However, the evidence more strongly supports the latter theory (Graf and Juto, 1994a; Graf and Juto, 1995). More recent studies utilising electron microscopy have indicated that RM may result in chronic inflammation and hypersecretion, due to hyperplasia of goblet cells and increased epidermal growth factor (Lin et al., 2004). Additionally, nasal turbinate samples from RM sufferers showed loss of ciliated cells and changes to vascular endothelial layer, via disruption of the basal lamina and increased interstitial gap (Knipping et al., 2007). These latter observations may explain the reduced mucociliary clearance and oedema experienced in RM (Knipping et al., 2007; Lin et al., 2004) and supports the previous findings from animal models. The role the inflammatory cascade plays in RM is also unclear. However, the reduced mucociliary clearance observed in RM may result in inflammatory mediators persisting at the nasal mucosa (Passàli et al., 2006). Furthermore, the proposed damage to the nasal epithelia may subsequently cause cell death. This damage has the potential to activate the inflammatory cascade and create a loop. Not only causing localised inflammation but also exposing tissue to pathogen-associated molecular patterns (PAMPs) leading to infiltration by leukocytes and neutrophils (Figure 2). However, for some individuals with RM there is also an element of underlying allergy. This may influence not only the commencement of nasal vasoconstrictors but also the prolonged use, and may explain some of the observations relating to inflammatory response (De Corso et al., 2020).

All of the hypothesised mechanisms have the potential to play a role in the pathophysiology of RM. Of particular interest are mechanistic changes to the innervation pathway either via negative feedback loops or degeneration of the nerve fibres, which may explain why some individuals suffer with RM. The morphological changes such as damage to ciliated cells, changes to secretions, oedema and changes to the endothelial layer, may explain the symptoms experienced during RM. Furthermore, the inflammatory cascade may play a role in causing changes to morphology and feed into changes in the innervation pathway. Thus, the summarised hypotheses would benefit from further *in vivo* studies to better determine the role they play in RM.

### 2.3 The role of imidazolines in rhinitis medicamentosa

The wide variety of topical nasal treatment available on the market, reflects the differences in the mode of action each provides. Furthermore, combining different types of nasal treatments may work synergistically, providing better relief for more persistent symptoms (Table 4) (Wang et al., 2024). The effectiveness of the sympathomimetics, and more specifically those which contain imidazoline derivatives, has made them one of the most widely available and utilised treatments for nasal congestion (Trinh et al., 2024). Sympathomimetics act by constricting the blood vessels in the nasal passageway relieving related congestion by reducing blood flow to the area (Wang et al., 2024). Part of the success of imidazoline derivatives is linked to their tolerability, with side effects experienced typically being minor when used correctly. Consequently, most individuals chose not to discontinue the product, as the benefits of treatment outweigh the minimal side effects. However, the limited side effects and overall good tolerability, relies on the patient taking the recommended dosage, for the maximum recommended time, to limit the likelihood of developing RM later on (Graf and Juto, 1995; Passàli et al., 2006; Chodirker, 1981).

**TABLE 4:** Comparative examples of different over-the-counter topical nasal treatments including onset and duration of action, target of treatment in respect to efficacy and relationship to rhinitis medicamḥentosa.

Variation in treatment effect	Corticosteroids	Antihistamines	Sympathomimetics	
			Imidazoline derivatives	β-phenylethylamine derivatives
Target of treatment	Allergic rhinitis Non-allergic rhinitis Sinusitis (chronic) Post-nasal drip Nasal Polyps	Allergic rhinitis	Nasal congestion due to Allergic rhinitis Non-allergic rhinitis Sinusitis Upper respiratory tract infections	Nasal congestion due to Allergic rhinitis Non-allergic rhinitis Sinusitis Upper respiratory tract infections Ear pressure
Mode of action	Reduce inflammation	Reduce irritation caused by allergens	Reduce congestion by vasoconstriction	Reduce congestion by vasoconstriction
Duration of action*	Up to 24 h	12–24 h	Up to 12 h	2–3 h
Onset of action*	6–12 h (initial effect) 1–2 weeks (for maximum effect)	15–30 min	5–10 min (subjectively from 25 s)	<5 min
Frequent reapplication required	No	No	No	Yes
Maximum length of use*	Long term (with medical guidance)	Long term (with medical guidance)	3–10 days	3–7 days
Causes rhinitis medicamentosa	No	No	Yes (if overused)	Yes (if overused)

*Time points vary depending on API used (Wang et al., 2024; Berlin et al., 2000; Russo et al., 2023; Graf and Juto, 1995; Eccles et al., 2008; Passàli et al., 2006; Chodirker, 1981; Schafer et al., 1992; Bucaretchi et al., 2003; Druce et al., 2018; Eccles et al., 2010; Graf et al., 2018; Hutcheon et al., 1955; Neistadt, 1955; Putnam and Herwick, 1946; Reinecke and Tschaikin, 2005; Steinberg, 1953; Wenzel et al., 2004; Burnham, 1932; Holm et al., 1999; Laekeman et al., 2010).

Imidazoline based nasal treatments have been available since the 1940s (the history of which is discussed in more detail in the first part of our review series), the early-generation imidazolines, for example, naphazoline, had an onset of action of approximately 5–10 min and they lasted 6–10 h depending on the derivative used. However, developments around the efficacy of this class of drugs has produced new-generation imidazolines, notably oxymetazoline, which provides significant subjective and objective relief for up to 12 h, with very rapid subjective onset of action. The improved symptom relief period equates to fewer doses per treatment being required, helping to minimise the total amount of imidazoline used which increases tolerability and helps to reduce potential side effects (Wang et al., 2024; Eccles et al., 2008; Schafer et al., 1992; Bucaretchi et al., 2003; Druce et al., 2018; Eccles et al., 2010; Graf et al., 2018; Hutcheon et al., 1955; Neistadt, 1955; Putnam and Herwick, 1946; Reinecke and Tschaikin, 2005; Steinberg, 1953; Wenzel et al., 2004). A potential risk that occurs whilst taking imidazoline based nasal treatments is the development of RM. The chance of which increases with prolonged use, thus for the majority of OTC preparations, maximum length of treatment is typically restricted, with variations depending on the formula used (Wang et al., 2024; Bridgeman, 2017; Jain, 1994).

Interestingly, although not directly linked to RM, the method used to deliver the treatment has the potential to help reduce the risk of its development. For example, metered-dose nasal sprays reduce the likelihood of dose variability when compared to nasal drops, and improves the drug delivery within the nasal passages. Consequently, patients feel like they are receiving an adequate dose and may feel less inclined to use extra doses, thereby potentially reducing the chance of RM developing (Bharagava et al., 2025). However, further studies are necessary to determine whether mode of delivery can quantifiably alter the risk of developing RM. In terms of reducing risk, additional studies centred around the minimal effective concentrations of various nasal vasoconstrictors may also be beneficial. This could help identify whether lower concentrations could continue to provide a similar decongestive effect but with a reduced risk of developing RM.

Whilst all sympathomimetics have the potential to cause RM, the improved efficacy of the imidazolines compared to the β-phenylethylamines means the risk is reduced however, this strongly relies on following dosage instructions correctly (Wahid, 2023; Davis and Eccles, 2004; Dushay and Johnson, 1989). However, to what extent patients adhere to usage instructions is unclear. Owing to the ease of access to OTC preparation, patients often have limited interactions with pharmacy or healthcare professionals (Russo et al., 2023; Mehuys et al., 2014). Furthermore, one study suggests that over 70% of patients self-medicate OTC imidazoline nasal vasoconstrictors. Consequently, this lack of interaction meant that 60% of patients were unaware of the risk associated with incorrect use (Khan et al., 2025). Therefore, it is not surprising that a second study estimated 40% of patients use higher than the recommended dosages and over 30% use nasal vasoconstrictors for longer than recommended (Russo et al., 2023). Given the high percentage of individuals who are unaware of the risk of incorrect use, coupled with the large number of patients who appear to misuse nasal vasoconstrictors, further education would be beneficial. Although patient education requires further improvement, the reduced risk afforded by the improved efficacy and tolerability of new-generation imidazoline derivatives has resulted in them becoming the decongestant of choice for many manufacturers and patients alike, with oxymetazoline being used most widely in the United States (Trinh et al., 2024).

### 2.4 The role of preservatives in rhinitis medicamentosa

One of the most widely used preservatives is BKC (Bharagava et al., 2025). BKC has extensive applications in pharmaceuticals, healthcare, personal hygiene products and cleaning products owing to its antiseptic and broad-spectrum antimicrobial properties. However, as a recognised skin and eye irritant, plus potential skin sensitiser, the maximum safe concentration in nasal decongestants is 0.1% (Davis and Eccles, 2004). Whilst the addition of BKC to nasal decongestants prevents bacterial contamination and extends shelf life, possible effects on the nasal mucosa and the potential impact this poses for the development of RM remains a topic of debate. For example, in healthy volunteers treated with oxymetazoline with or without BKC, 3 times daily for 30 days, although rebound swelling occurred in both groups, the effect was significantly worse in the group exposed to BKC (Schäfer et al., 1995). Furthermore, the effects seem to persist beyond the cessation of the initial treatment. Repeated exposure for 10 days, having 3 months prior been treated with the same combination for 30 days, resulted in increased nasal stuffiness being experienced after 4 days of treatment and increased swelling of the nasal mucosa after 10 days. Conversely, treatment with oxymetazoline alone, on both occasions, did not cause a similar extent of swelling or stuffiness after 10 days of treatment (Dushay and Johnson, 1989). A similar study also showed that BKC when used in isolation resulted in some swelling of the nasal mucosa. Whilst an increased sense of nasal stuffiness and nasal reactivity was experienced when oxymetazoline was used in isolation. Thus, BKC may have an additive effect when combined with nasal vasoconstrictors and used excessively, further enhancing the sensation of nasal stuffiness experienced by increasing nasal swelling (Mehuys et al., 2014). Whilst not directly related to the development of RM, treatment of healthy individuals with saline nasal spray containing BKC has been shown to impair mucociliary clearance after 3 weeks of use (Khan et al., 2025). Similarly, an *in vitro* study on nasal epithelial cells showed BKC inhibited ciliary beat in a dose dependent manner, which may explain the observation around impaired mucociliary clearance. However, this latter observation comes with a caveat that *in vitro* studies do not completely mimic the *in vivo* environment, the lack of mucus *in vitro* may explain why this observation does not translate to similar findings in human volunteers (Marple et al., 2004).

As with many other aspects of the discussion around RM, the role BKC plays is contradictory. A study on primary human nasal epithelial cells measuring saccharin transport time and levels of cytokines and inflammatory cells, showed that *in vitro* BKC was ciliotoxic but, *in vivo* equivalent observations could not be confirmed (Merchel Piovesan Pereira and Tagkopoulos, 2019). The observation of no change in ciliary transport time has also been considered alongside changes to cellular ultrastructure, which after 6 weeks treatment with BKC alone or in combination with a glucocorticoid steroid nasal spray remained unchanged (European Medicines Agency). Furthermore, a review of 18 studies indicated that BKC used at a maximum concentration of 0.1% in intranasal products appeared to be safe with minimal evidence of statistically significant differences between BKC treated and control groups (Marple et al., 2004). Although BKC has been classified as safe at the therapeutic dose used, there is some evidence to suggest that while it may not be directly responsible for RM, it can aggravate the symptoms. In addition, it may also cause irritation to the nasal mucosa and result in damage to ciliated cells. However, to better determine whether it has an effect future research studies directly comparing incident rates of RM in preservative free preparations *versus* preservative containing preparation in human volunteers would be beneficial. Part of the current issue with understanding the role preservatives play in RM compared to preservative free formulations is that the presence of BKC is not always clearly defined. Thus, it is imperative that in future studies, the presence or absence of preservatives are clearly identified to ensure that their role can be more fully determined.

Regardless of the outcome of such future studies, a move towards preservative free treatments may help further alleviate RM symptoms and would ultimately be in the interest of the patient. Such a change would likely have implication for the regulatory guidelines for the current approved maximum concentration of BKC in intranasal products (Merchel Piovesan Pereira and Tagkopoulos, 2019). The European Medicines Agency identifies the current average concentrations of BKC in nasal preparations as 0.02–0.33 mg/mL. Furthermore, they state that no safe clinical threshold can be established based on current research (European Medicines Agency). In fact, in Europe the use of BKC in antiseptic hand and body washes for personal use is no longer approved and the residual levels in food products have been reduced. The same is not true for the United States (Merchel Piovesan Pereira and Tagkopoulos, 2019). Thus, further research around the role of BKC in RM may help to establish a safe clinical threshold for its used in nasal vasoconstrictors. This could enable regulatory guidelines to be altered to better reflect any impact it has on nasal mucosa health. However, this shift is complicated by the need to manage microbial contamination and product stability without compromising effectiveness of treatments. A proposed alternative to managing microbial contamination without a preservative is the use of an acidified solution. In a short-term trial, the acidified solution appeared to be effective at preventing microbial contamination and was tolerated as well as BKC (Ryan and Hwang, 2010). Interestingly, the use of acidified solutions may also help improve stability of the oxymetazoline used in nasal vasoconstrictors (Stanisz, 2002). Furthermore, the addition of, e.g., dexpanthenol to oxymetazoline or xylometazoline nasal vasoconstrictors may mitigate some of these issues, whilst providing other benefits (Jagade et al., 2008).

### 2.5 Development of tolerance and addiction to imidazoline based nasal treatments

**FIGURE 3:**
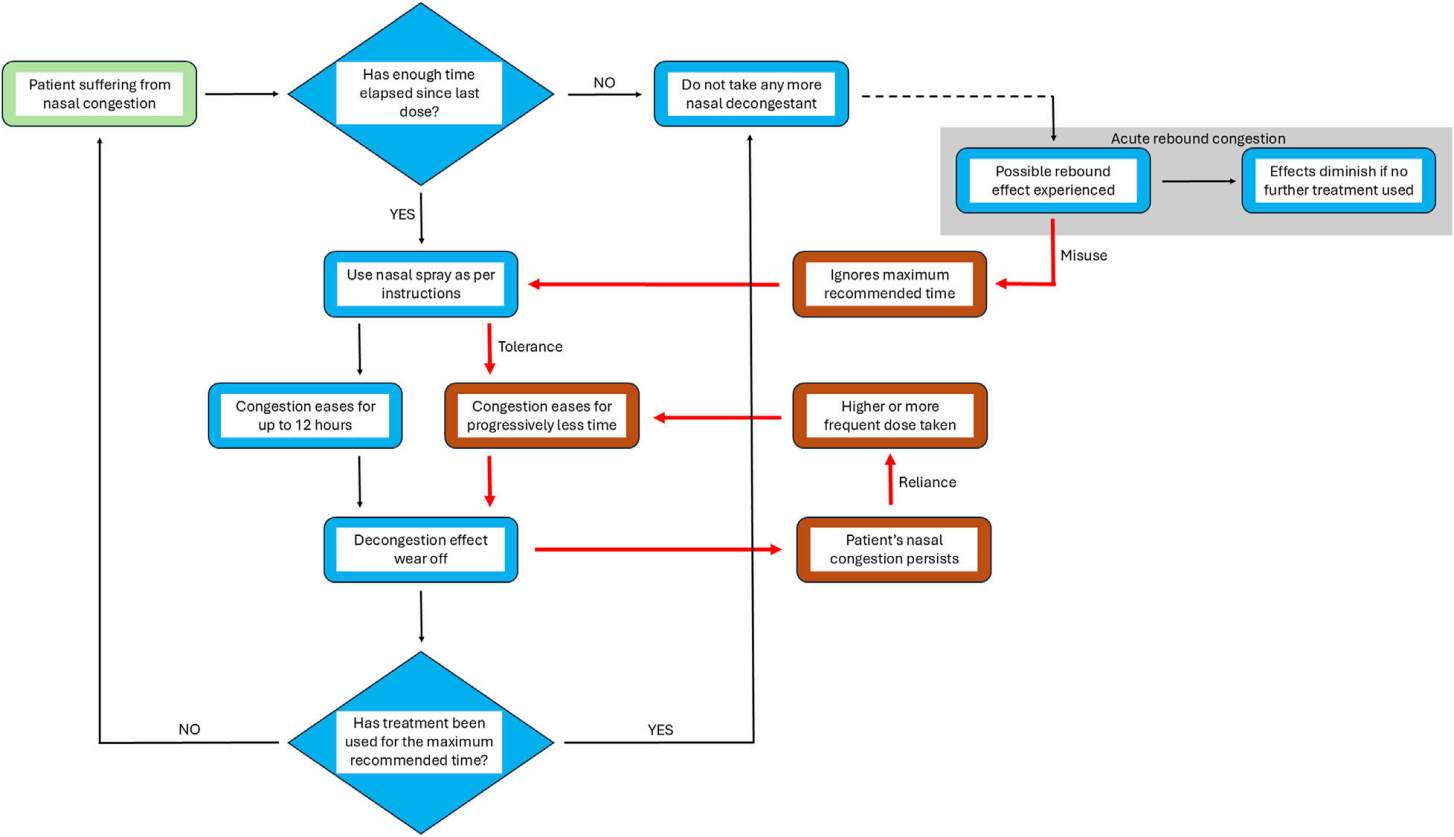
From relief to reliance: the prolonged use cycle of nasal vasoconstrictors. Model representing the normal stages of nasal decongestant spray use and the changes which occur during the cycle of prolonged, excessive use. Black arrows represent the stages in the process of treating nasal congestion from the point of recognition of symptoms (identified by green box) to the resolution of the condition after treatment with nasal vasoconstrictors as recommended. Red arrows (and boxes) represent the stages in the process when misuse/overuse leads to a rebound state, finally resulting in rhinitis medicamentosa. In cases where after completing the treatment acute rebound congestion occurs patients might continue with treatment, beginning a cycle of misuse of the nasal vasoconstrictor. If this initial misuse use of treatment is prolonged, congestion is eased for progressively shorter periods of time as tolerance develops and a higher dose is required to achieve the same effect, resulting in the development of rhinitis medicamentosa. Thus, a cycle of reliance is entered resulting in prolonged and excessive use of nasal vasoconstrictors without considering the time elapsed between treatments or length of time treatment is used for.

The reason why and how RM develops is not fully understood. However, there is a wealth of information suggesting that the prolonged use of imidazoline based nasal treatments plays a significant role, regardless of the mechanisms involved. The reason why individuals begin to take nasal vasoconstrictors excessively is potentially linked to the development of tachyphylaxis to the API in use. Development of a tolerance towards an imidazoline based nasal treatment results in a shortening window of relief with each dose. With xylometazoline, for example, the effectiveness of the treatment is reduced from 9 h to around 5 h after 30 days of use. This reduction in efficacy is linked to a potential reduction in affinity to the α-adrenergic receptors or a downregulation in their expression rather than a specific tolerance (Schäfer et al., 1995; Zheng et al., 2025). This development of tachyphylaxis to the API in nasal vasoconstrictors is a common experience for many patients. To continue feeling relief from nasal congestion, they increase the frequency and/or volume of dose, creating a cycle whereby their symptoms continue to deteriorate although they are being treated. This resulting prolonged use of nasal vasoconstrictors is potentially due to a lack of awareness by patients regarding the risk of developing RM if recommended limits are not followed. Thus there is little recognition that the cycle which is entered as a consequence of misuse is detrimental to their symptom relief (Eccles et al., 2008) (Figure 3).

The prolonged use of sympathomimetic nasal treatments has been linked to the concept of addiction through misuse. The prevalence of abuse of OTC medication is not known. However, it is widespread globally and is more problematic in young adults and adolescents compared to other age groups. The potential for developing an addiction may reflect how rapidly symptoms of nasal congestion are relieved, thus individuals take higher doses for longer than is recommended to provide continued relief (Bhargava et al., 2011; Ryan and Hwang, 2010; Stanisz, 2002). This addiction to nasal vasoconstrictors, particularly imidazoline derivatives, is sometimes termed ‘privinism’. The idea concerns the decline in effectiveness of treatment which occurs through prolonged use, which results in a continued sensation of congestion, when previously it has been reduced. The reduction in effectiveness leads to a cycle whereby, the patient increases the dose taken or frequency of dose as they endeavour to continue to relieve their nasal congestion symptoms (Schäfer et al., 1995; Lin et al., 2004; Morissette et al., 2007; Jagade et al., 2008). The likelihood of becoming addicted to nasal vasoconstrictors may stem from the seeming unawareness of potential side effects. Of the individuals who regularly use nasal vasoconstrictors, as few as 20% may be aware of adverse effects, while only a quarter of regular users are aware of the potential for addiction and the recommended maximum period of treatment (Bhargava et al., 2011; Ryan and Hwang, 2010). Interestingly, even discontinuing use of the decongestant for over 12 months in patients who had previously suffered from RM does not return patients to a similar level of risk as those individuals who have never suffered. In these individuals rebound swelling occurred rapidly when oxymetazoline was used 3 times a day, for 7 days. Thus, individuals who have previously used imidazoline based nasal treatments excessively or for a prolonged period, but have successfully withdrawn treatment, need to consider the potential risk of developing RM quickly if they begin to use them again, even at the recommended dose and frequency (Lakatos et al., 2025).

Physical dependence to nasal vasoconstrictors is primarily born out of the development of tachyphylaxis to the API in use, rather than any psychoactive effect. However, there also appears to be a certain element of psychological dependence to this addictive behaviour. This often centres around a patient’s need to feel they can breathe freely through the nose. Which when absent can result in feeling of anxiety, sleep disturbances and a sensation of suffocation. Nevertheless, when dealing with patients who are dependent on nasal vasoconstrictors both aspects need to be considered (Lakatos et al., 2025). Given the lack of patient awareness around the risk of adverse side effects and potential for addiction whether this be physical or psychological, improvements in patient education need to be addressed. The ease of access of nasal vasoconstrictors makes addressing this problematic, as information leaflets alone are unlikely to improve patient’s adherence to recommendations (Barrias et al., 2024). As such, public health campaigns and pharmacist-led interventions may be better placed to improve patient knowledge and provide a more comprehensive overview of the risk associated with the misuse of nasal vasoconstrictors.

## 3 Treatment and prevention of rhinitis medicamentosa

As nasal congestion can have a significant physiological impact, affecting breathing, sinus pressure, sleep quality, sensory function and overall health. Addressing nasal congestion promptly is essential to restoring normal physiological function and preventing complication. Furthermore, given that the impact and burden of nasal congestion felt globally, the widespread use of nasal decongestants is inevitable. 1t is worth noting that there is no standardised medical treatment method for RM, which may reflect the lack of consensus relating to the pathophysiology of the disease. However, any treatment received is typically beneficial and successfully reduces the symptom burden. Furthermore, education of the patient around the cause of their rhinitis and the risk associated with the prolonged use of nasal vasoconstrictors, plays an important role in helping to prevent the development of RM from the outset (Scadding, 1995; Zucker et al., 2019; Stewart et al., 2010; Meltzer et al., 2009). Education around RM and rhinitis in general is lacking, thus it is unsurprising that an individual suffering with chronic nasal congestion may continue to take a nasal decongestant beyond the recommended limit as tolerance to the treatment begins to occur. This prolonged use of nasal vasoconstrictors inevitably results in the development of RM, which without intervention is unlikely to resolve itself.

To effectively manage the treatment of RM, it is imperative that symptom burden is assessed both before and after treatment. This is most frequently done using the Sino-Nasal Outcome Test-22 (SNOT-22). Designed to assess the burden of symptoms on the patient’s quality of life, it considers both the physiological and psychological impact (La et al., 2023). However, the psychological aspect of the questionnaire can be somewhat subjective in nature. Consequently, if this played a large part in their symptom burden, score may improve but less than anticipated. Thus, whilst it provides both a relatively holistic view of symptom burden and a comparative measure of effectiveness of treatment, it should not be considered in isolation but used alongside objective measurements of nasal obstructions (Koskinen et al., 2021; Gallo et al., 2020).

**FIGURE 4:**
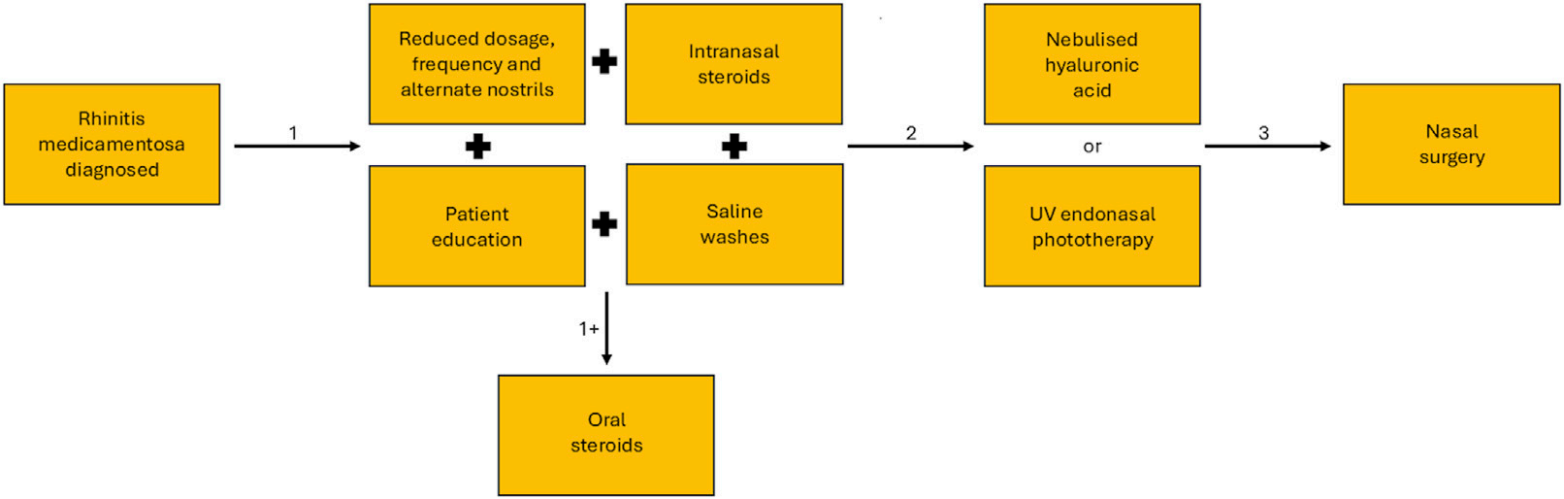
Treatment pathway for the resolution of symptoms of rhinitis medicamentosa. After initial diagnosis conservative methods of treatment (1) are used, this begins with phased weaning and may include saline washes, intranasal steroids and patient education, in some cases adjunct oral steroids may be required (+1). If conservative methods of treatment fail, alternative treatment methods may be tried (2) including nebulised hyaluronic acid and UV endonasal phototherapy. If less invasive methods are unsuccessful, the surgical intervention may be required.

RM is typically a chronic condition, which is reversible given a suitable clinical approach and treatment to support the withdrawal of the nasal decongestant. However, with no formal clinical guidelines or standardised approach to treatment, other than cessation of the nasal vasoconstrictor, the choice of treatment may vary (Fowler et al., 2019). Treatment involves the slow withdrawal of the nasal decongestant, as sudden cessation can cause further rebound congestion, accentuating symptoms and inducing further misuse. This weaning period is carried out in conjunction with intranasal steroids, which aid in reversing the tolerance developed at the α-adrenergic receptors and associated rebound congestion, saline washes to help reduce symptom burden, and if required adjunct oral steroids (Fowler et al., 2019; Zheng et al., 2025; Vaidyanathan et al., 2010). When conservative approaches are unsuccessful alternative treatments can include surgical procedures to improve airflow through the nasal cavity (Margulis et al., 2024). Nebulised hyaluronic acid to aid regeneration and repair of the nasal mucosa and regulation of mucociliary clearance (Bergonzani et al., 2023; Casale et al., 2017). Or the application of UV endonasal phototherapy to reduce inflammation in the nasal cavity (Figure 4) (Carson and Lyons, 2019). Although RM has the potential to cause a notable symptom burden for the patient, especially given its chronic nature, with suitable medical intervention and treatment management the reliance on nasal vasoconstrictors can be removed and the patient can return to a life without nasal congestion.

Whilst the treatment of RM is typically successful with medical or in extreme instances surgical intervention, it can be lengthy and problematic for the patient. Thus, the use of sympathomimetic nasal treatments should come with a caveat that prevention is the best cure where RM is involved. Therefore, it is imperative that clear and concise information around how the correct use of a topical nasal treatment can optimise the delivery and benefit of each application is provided to the patient. The optimisation of delivery needs to consider several different elements, such as clearly highlighting the maximum length of time a specific treatment can be used for and risk of not following the recommended dose. Additionally, considering the duration of symptoms relief provided by a specific API, could result in less frequent dosing, whilst good efficacy ensures lower dosages over time. Furthermore, if both the subjective and objective relief experienced by the patient is rapid and long-lived, then this may help reduce the likelihood of entering the vicious cycle of taking more frequent or larger doses, to try relieving symptoms which are aggravated by taking excessive amounts of the treatment for prolonged periods. With this in mind, improving the education patients receive around the use and misuse of nasal vasoconstrictors, has the potential to prevent them entering the cycle of misuse and create a noticeably decrease in the cases of RM. However, barriers to patient education currently exist. Patient education and advice typically take the form of information leaflets. Whilst many patients read them when first using medication, they are unlikely to check subsequent packets, thus missing updated information. Furthermore, the technical language, volume and relevance of information provided can make these leaflets difficult to access (Barrias et al., 2024). As such, making it a necessary requirement to seek advice from a healthcare professional prior to purchasing nasal vasoconstrictors would ensure patients are educated in the risks associated with taking this medication and the impact of misuse. This has a two-fold benefit of reducing the symptom burden not only for themselves, but also reducing pressure on the healthcare systems which have to treat the consequences of this misuse (Scadding, 1995; Barrias et al., 2024; Tong et al., 2016).

## 4 Conclusion

Studying RM pathophysiology is difficult as it is not clear which of the observed symptoms are caused by the underlying disease or nasal vasoconstrictor misuse. The development of RM appears to be the culmination of a series of phases which arise through the misuse of nasal vasoconstrictors. In the correct-use-phase, nasal vasoconstrictors are considered safe and the risk of developing any rebound congestion is minimal. However, vasodilatation can occur after treatment cessation, prompting some patients to begin misusing the nasal vasoconstrictor, thus entering the rebound phase. As this misuse continues, relief is provided for progressive shorter intervals, resulting in larger or more frequent doses, thus entering a period of prolonged use culminating in the RM phase. Once chronic RM has developed then the patient is likely to require further intervention to withdraw treatment of the nasal vasoconstrictor. Thus, there is a need to understand the mechanisms involved in development of RM, so that the burden of symptoms for both the patient and healthcare provider can be lessened. The impact that differing formulations have on nasal mucosa health when used excessively, is not fully understood. Further, whether this is a direct consequence of the drug, the addition of preservatives in the treatment or the pathophysiology of RM is difficult to determine from the current literaturehowever, it is worth considering how the risk of developing RM can be mitigated against. Patient education should form a major part of this mitigation, with a move away from relying on information leaflets, towards more pharmacist-led interventions or public health campaigns. Which may provide a greater opportunity to influence patient choice, providing a mechanism to share valuable information regarding the correct use of nasal vasoconstrictors, the risks associated with misuse and when to seek further medical advice. In addition, considering a move towards preservative free formulations would remove one possible contributor of RM, whilst taking a less-is-more attitude to frequency and strength of dose may still provide symptom relief but further minimize the risk. Further, where RM is concerned prevention may just be the best medicine. With this in mind and given the gaps within the literature, it would be beneficial to not only investigate the mechanisms involved in RM, but also consider the use of multi-centre studies to provide greater clarity around the differing individual responses to nasal vasoconstrictors. Consequently, this may give rise to improved patient-oriented treatment management plans, to help reduce reliance on nasal vasoconstrictors even at the acute rebound stage, before RM develops.
